# Nutritional Value of Yogurt as a Protein Source: Digestibility/Absorbability and Effects on Skeletal Muscle

**DOI:** 10.3390/nu15204366

**Published:** 2023-10-14

**Authors:** Koichiro Sumi, Ryoichi Tagawa, Kae Yamazaki, Kyosuke Nakayama, Takefumi Ichimura, Chiaki Sanbongi, Koichi Nakazato

**Affiliations:** 1 Nutrition and Food Function Research Department, Food Microbiology and Function Research Laboratories, R&D Division, Meiji Co., Ltd., Nanakuni, Hachioji 192-0919, Japan; 2 Next Generation Monozukuri Research Department, Food Science & Technology Research Laboratories, R&D Division, Meiji Co., Ltd., Nanakuni, Hachioji 192-0919, Japan; 3 Department of Exercise Physiology, Nippon Sports Science University, 7-1-1 Fukasawa, Setagaya-ku, Tokyo 158-8508, Japan; nakazato@nittai.ac.jp

**Keywords:** yogurt, milk protein, digestion, absorption, skeletal muscle, fermentation, lactic acid bacteria

## Abstract

Yogurt is a traditional fermented food that is accepted worldwide for its high palatability and various health values. The milk protein contained in yogurt exhibits different physical and biological properties from those of non-fermented milk protein due to the fermentation and manufacturing processes. These differences are suggested to affect the time it takes to digest and absorb milk protein, which in turn will influence the blood levels of amino acids and/or hormones, such as insulin, and thereby, the rate of skeletal muscle protein synthesis via the activation of intracellular signaling, such as the mTORC1 pathway. In addition, based on the relationship between gut microbiota and skeletal muscle conditions, yogurt, including lactic acid bacteria and its metabolites, has been evaluated for its role as a protein source. However, the substantial value of yogurt as a protein source and the additional health benefits on skeletal muscle are not fully understood. The purpose of this review is to summarize the research to date on the digestion and absorption characteristics of yogurt protein, its effect on skeletal muscle, and the contribution of lactic acid bacterial fermentation to these effects.

## 1. Introduction

### 1.1. What Is Yogurt?

Yogurt is consumed in many cultures worldwide as a dessert and culinary ingredient because of its high palatability and nutritional value. Yogurt is produced mainly by fermenting cow milk via lactic acid bacteria and is defined in the International Food Standard (CODEX) as “milk or dairy products obtained by lactic acid fermentation by the action of *Streptococcus thermophilus* and *Lactobacillus delbrueckii* subsp. Bulgaricus” [1,2]. The production of yogurt involves various processes, such as homogenization, heat treatment (pasteurization), fermentation, and smoothing. Depending on the combination of these processes, various types of yogurts with completely different physical properties can be produced. For example, there is set yogurt, which is produced by coagulating the casein micelle by lowering the pH due to lactic acid fermentation after it is placed in a container; stirred yogurt, which is produced with a smooth texture by stirring after fermentation; drinking yogurt, which is transformed into a fluid by shearing after fermentation and stabilized with stabilizers; and concentrated yogurt, typically named “Greek style”, manufactured by straining after fermentation. In addition, even within the same type of yogurt, the physical and/or chemical properties, such as viscosity and hardness, can vary by changing the manufacturing conditions [3].

### 1.2. Benefits of Yogurt for Human Health

Yogurt has also been recognized as a functional health food in many biological aspects. As one of the most representative probiotics, yogurt has been reported to influence the composition of the intestinal flora by reducing pathogenic bacteria [3]. Other diverse benefits for human health have been reported for yogurt, such as intestinal regulation, immunomodulatory action, diabetes prevention, and cardiac disease prevention [4]. Furthermore, its nutritional value should also be noted. Yogurt is a source of various vitamins and minerals. In particular, yogurt is a good source of calcium because its absorption is facilitated by galacto-oligosaccharides and casein phosphopeptides in dairy products [5]. Yogurt, like milk, also contains high levels of short-chain fatty acids [6] and various milk protein-derived peptides, and it has been suggested that these components of yogurt help reduce intestinal inflammation and protect the immune system [7]. In addition, a large cohort study from 21 countries reported that consumption of full-fat dairy products, including yogurt, lowers the risks of metabolic syndrome, hypertension, and diabetes [8].

### 1.3. Physical/Biological Properties and Digestion/Absorption of Milk Protein

Yogurt is also important in that it is rich in protein. The major protein sources with higher bioavailability include milk, meat, fish, eggs, and soybeans, among others. Milk protein is rich in essential amino acids for humans and has a very good ratio at which it is absorbed and taken up into the body. To reflect this, the digestible indispensable amino acid score of milk protein is particularly superior among these major protein sources [9,10,11]. Milk protein is broadly divided into casein and whey proteins. Traditionally, when cheese is produced from milk using rennet, the protein in the solidified part of the cheese body is casein, and the protein in the residual water, called whey, is whey protein [12]. Total milk protein is composed of casein and whey proteins at an approximate 8:2 ratio. Casein proteins consist of alpha-, beta-, and kappa-casein, and these caseins are present in a uniform colloidal dispersion in milk by forming micelles together with calcium phosphate [13]. Whey protein is composed of approximately 50% β-lactoglobulin (β-Lg), 20–25% α-lactalbumin (α-La), and 25–30% other proteins, including serum albumin, immunoglobulin, and lactoferrin; these whey proteins are water soluble [14]. As mentioned above, whey is a by-product of cheese production, but whey proteins have a high content of essential amino acids, such as branched-chain amino acids (BCAAs), giving whey its high nutritional value. Therefore, these proteins are refined as whey protein concentrate or whey protein isolate, which are used as protein sources. β-Lg is rich in BCAAs, among other proteins, but is potentially the cause of bovine milk allergy because it is not present in human breast milk [15].

When milk proteins ingested from the mouth reach the stomach, casein is broken down by pepsin digestion and gastric juice, which has a very low pH, resulting in agglomerates and precipitates as large casein particles, and these particles remain in the stomach for a long time. On the other hand, whey protein is nearly unaffected by pepsin or very low pH, and it is gastrically excreted to the small intestine over a short time [16]. In particular, β-Lg is highly resistant to pepsin and passes almost undigested through the stomach [14]. However, all of the proteins, including the pepsin-undigested whey proteins, are neutralized in the small intestine by pancreatic juice and then digested by enzymes in the pancreatic juice, including trypsin, chymotrypsin, and pancreatic elastase [17], into oligopeptides or amino acids [18]. Residual oligopeptides are degraded into amino acids by endoaminopeptidases expressed in small intestinal epithelial cells [17]. Amino acids are absorbed into the portal vein from the small intestine via various amino acid transporters for neutral amino acids, cationic amino acids, specific amino acids, and others present in the small intestine [19]. Moreover, di- and tripeptides are absorbed via peptide transporters [19].

Because protein digestion and absorption proceed rapidly in the small intestine after gastric emptying, gastric emptying dynamics are considered a major factor determining the rates of digestion and absorption of milk proteins [20]. Differences in the rate of digestion/absorption of proteins are reflected by blood amino acid dynamics, such as the timing and degree of the increase, peak, maintenance, and decrease in blood amino acid concentrations [21]. As mentioned above, casein has a longer residence time in the stomach, while whey protein’s residence time is shorter. Reflecting at least this property, it has been shown in humans and animals that blood amino acid concentrations rise faster during whey protein ingestion than during casein ingestion [22,23]. However, even when compared with whey protein, the rate of increase in blood amino acid concentrations is faster when enzymatically pre-degraded whey peptides are ingested [24]. These findings suggest that the digestibility of the protein itself and prior partial digestion may also affect the dynamics of blood amino acids.

### 1.4. Milk Protein and Skeletal Muscle Protein Synthesis

Skeletal muscle quantity and quality are important for healthy living in all people, as skeletal muscle is associated with not only improved performance in athletes [25,26] but also reduced risk of various diseases [27], maintenance of locomotor function in the elderly [28] and prolonged life span [29]. Skeletal muscle mass is specified by the net balance between muscle protein synthesis (MPS) and muscle protein breakdown (MPB) [30]. In other words, if the total MPS exceeds the total MPB within a time period, skeletal muscle mass will increase, although MPS and MPB are constantly fluctuating. One of the greatest factors affecting MPS/MPB is dietary (protein) intake, with low MPS and high MPB before meals, but high MPS and low MPB after meals, and this diurnal variation is repeated daily [31]. The increase in MPS and decrease in MPB after protein ingestion are due mainly to the increase in blood insulin and amino acid concentrations after a meal [32]. Insulin is a hormone that promotes glucose intracellular uptake from blood [33], but it also contributes to the increase in MPS and decreases in MPB. When insulin acts on insulin receptors in skeletal muscle cells, it activates the intracellular PI3K/Akt pathway, involving translocation of the glucose transporter Glut4 from inside the cell to the plasma membrane, resulting in increased glucose uptake into skeletal muscle and decreased blood glucose levels.

Akt also affects mTORC1, a kinase complex acting as a master regulator of protein synthesis [34], and FOXOs, transcription factors that control proteolysis via the ubiquitin–proteasome system [35]. Akt leads to the activation of mTORC1, inducing protein translation initiation and elongation via phosphorylation of the mTORC1 target proteins p70 S6K and 4E-BP1, and results in the elevation of MPS [36]. In addition, Akt directly phosphorylates FOXOs, thereby inhibiting their nuclear translocation [35]. FOXOs are transcription factors for Atrogin1 [37] and MuRF1 [38], which are ubiquitin ligases necessary for proteolysis, and Akt decreases MPB by suppressing their transcription. Insulin is secreted from the pancreas in response to the blood glucose level [33] and protein intake level [39]. Insulin elevation after protein intake is due to the secretion of gastrointestinal hormones, such as GLP-1 and GIP [40], and to the elevation of blood amino acids [41,42]. Therefore, insulin elevation is a mechanism of increasing MPS and decreasing MPB after protein intake.

The other major mechanism is due to specific amino acids supplied from food proteins that act as direct stimuli to activate MPS. The most well-studied is the BCAA leucine, which can activate mTORC1 in a PI3K/Akt-independent manner via the Sestrin2/GATOR2- and leucyl/tRNA synthase-mediated pathways [43], although it also affects PI3K/Akt mediated mTORC1 activation [44]. It has also become clear in recent years that other amino acids have a role in enhancing MPS [45]. Of course, since amino acids are the building blocks of nascent proteins, an adequate supply of all amino acids is necessary to increase skeletal muscle mass.

Besides diet, exercise has a major influence on muscle protein metabolism [46]. Resistance exercise (RE), commonly referred to as muscle training, especially increases both MPS and MPB [47]. During RE, if adequate protein supplementation is provided, MPS increases additively, and the skeletal muscle protein net balance tilts significantly toward positive [48]. It is also important to note that the effect of RE lasts longer than traditionally assumed [49], with additional increasing postprandial MPS for 1–2 days after RE [50]. Therefore, with habitual RE, MPS exceeds MPB, and muscle hypertrophy occurs as an adaptation to muscle training. Thus, considering the optimal quantity and quality of protein from food to promote MPS should be an effective strategy for maintaining and improving muscle mass [51]. In addition, elderly and chronically ill patients exhibit anabolic resistance, which is desensitivity to anabolic stimuli, such as exercise and nutrition, and the causes of skeletal muscle loss, such as primary/secondary sarcopenia [52]. In other words, to achieve the same gains as younger people, they would have to consume larger amounts of protein than younger people. However, this is not a practical solution, and therefore, a protein source that can elevate MPS more effectively is needed.

Since an increase in blood amino acid concentrations, such as leucine, promotes MPS [53], not only the amount of protein ingestion but also its digestion and absorption properties affect postprandial MPS [54]. This “fast” protein source may be more effective in maintaining skeletal muscle compared with ”slow” protein sources, especially in the elderly [55]. Milk protein and its components, such as whey and casein, have been studied extensively [22,56] as representative high-quality protein sources. On the other hand, despite being a widely consumed yogurt since ancient times, its effectiveness as a protein source is still unclear, particularly for skeletal muscle health. The purpose of this review is to summarize the current evidence by focusing on the relationship between the digestion/absorption characteristics of milk proteins in yogurt and their effects on muscle synthesis and muscle mass and is to serve as a bridgehead for future research.

## 2. Digestion/Absorption Properties of Protein in Yogurt

### 2.1. Digestion/Absorption Effectors of Yogurt Protein

**Figure 1:**
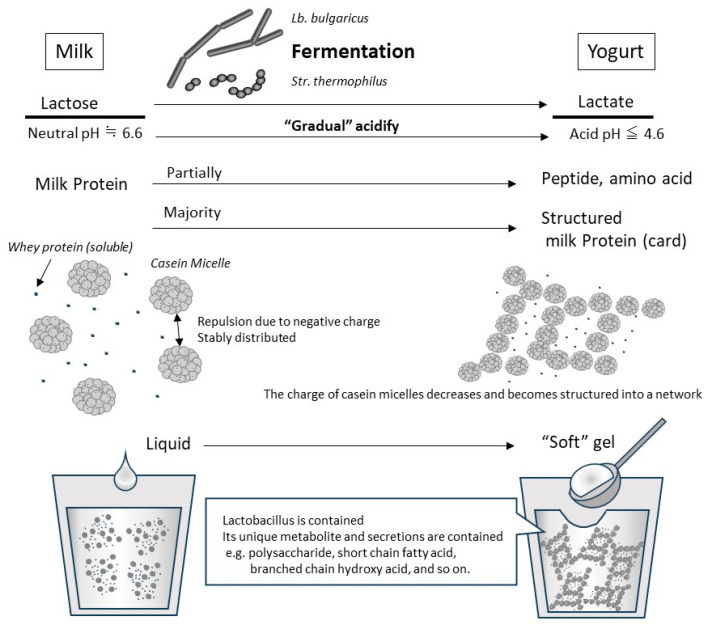
Typical changes due to lactobacillus fermentation at yogurt production from milk. An illustration of the changes during the production of “set yogurt”, which is produced by the most basic method of static fermentation. When the original milk is inoculated with *Lb. bulgaricus* and *Str. thermophilus*, they grow using lactose as a carbon source. In the process, lactose is converted to lactic acid, which decreases pH gradually. In fresh milk whose pH is neutral, casein micelles are negatively charged and are stably dispersed in the liquid due to their repulsion. As the fermentation progresses and the pH drops below 5.2, the casein micelles lose their charge and coagulate with each other. However, because this acidification and aggregation occurs slowly, casein micelles become a network structure that includes whey protein and water, so they are set as a soft gel (called card). By adding steps such as shearing and draining during or after fermentation, it is possible to create yogurt with different physical properties.

Yogurt contains whey and casein in roughly the same proportions as those in original raw milk, although some of the proteins are partially broken down by fermentation [57]. Approximately 90–99% of the whey protein in raw milk is denatured by heat treatment prior to fermentation (generally 5–10 min at 90–95 °C) [58], and denatured whey proteins, such as β-Lg, are combined with casein micelle [59]. In addition, the decrease in pH due to lactic acid fermentation causes the casein to approach its isoelectric point (approximately pH 4.5–4.2), resulting in a decreased electrical repulsive force among casein micelles (Figure 1). Because the pH-decreasing process in fermentation is gradual and slow, the casein micelles in yogurt do not coagulate and precipitate as hard as in the case of abrupt contact between raw milk and gastric acids in the stomach. In the yogurt fermentation process, the casein micelle particles are hydrated and form a homogeneous mesh-like structure, which becomes soft white tissue [13] (Figure 1). Because whey proteins that do not combine with casein micelles are soluble in this gradually decreasing pH range, they remain dissolved and present in trapped water in the above casein micellar network or in the released free water from the yogurt structure. Therefore, the physical properties of milk proteins in yogurt differ from those in raw milk, based mainly on the organization of the casein micelle during the production process.

Thus, the absorption rate of milk proteins may be higher in yogurt than in the original raw milk because milk proteins become frangible soft tissue at a pH below the isoelectric point of casein during the fermentation process. It was originally also considered that a portion of the milk protein is partially broken down during fermentation, which increases digestibility [60]. Thus, these properties have been expected to increase the protein absorption of yogurt. Indeed, *Streptococcus thermophilus* and *Lactobacillus delbrueckii* subsp. *bulgaricus*, which are used in yogurt production, have the ability to at least partially decompose and utilize casein [61], β-Lg, and α-La [62] as their amino acid sources. Reflecting this, it has been shown that peptides produced by artificial digestion are also different between yogurt and original raw milk [57]. However, in more recent artificial digestion studies using more physiological conditions, the digestibility of the protein in yogurt was more influenced by heating, such as during pasteurization, and by the milk protein composition of the raw milk [63] than by fermentation-based changes [64,65]. In macroscopic protein digestion, at least as measured by the increase in soluble protein and by the disappearance of major protein bands on SDS-PAGE, the influence of lactobacillus fermentation as the main factor is not clear. Also, as mentioned in Section 1.3, the physical properties of the protein-containing foods also affect digestion and absorption kinetics in vivo. Therefore, the differences in physical properties between yogurt and raw milk should be reflected by their digestion and/or absorption kinetics.

### 2.2. Slower Rate of Digestion/Absorption of Yogurt Protein Compared with Milk Protein

Various factors are involved in the digestion and absorption kinetics of proteins in yogurt. Reflecting this, in vivo studies have shown mixed results regarding how the digestion and absorption kinetics of milk proteins in yogurt differ from those in raw milk. In a study in which ^15^N-labeled milk or yogurt was given to miniature pigs, their digestion and absorption kinetics were examined based on the intestinal residue after 1, 2, 4, 8, and 12 h. As a result, the absorption of protein was faster during the first 2 h but slower after 2 h in raw milk than in yogurt. However, proteins in each were rapidly absorbed after reaching the intestines [20]. In a study conducted by the same group in adult males and females, the jejunal contents were continuously sampled through a nasogastric tube from before to 4 h after ingestion of ^15^N-labeled milk or yogurt. Similarly, in an animal study, the fastest rate of excretion from the stomach to the intestines was 20 min or 60 min after ingestion of milk or yogurt, respectively; both were rapidly digested and absorbed after reaching the proximal jejunum [66]. In a study in which young men ingested a test food mixed with ^13^C-labeled sodium acetate and their exerted ^13^CO_2_ in the breath was measured, Caspian Sea yogurt (strictly, this is not yogurt because it is fermented with *Lactobacillus acidophilus* and *Lactococcus cremoris*) took longer to digest/absorb compared to milk (195 vs. 110–150 min) [67]. In summary, those studies show that gastric emptying had the most important effect on the rate of digestion and absorption, and these results might be explained by yogurt’s longer gastric emptying because of its higher viscosity compared with milk.

### 2.3. Faster Rate of Digestion/Absorption of Yogurt Protein Compared with Milk Protein

On the other hand, in a study examining changes in blood total amino acid (TAA) concentration in elderly people over a 5 h period following consumption of various dairy products, the peak blood TAA concentration was higher, and the time to reach peak TAA concentration was shorter for yogurt ingestion compared with raw milk or cheese ingestion [68]. Moreover, using rat portal blood TAA concentrations as an indicator of protein digestion/absorption, we have consistently found that yogurt is more digestible and absorbable compared with original raw milk. In brief, in yogurt and original low-fat milk, which were pasteurized and homogenized under identical conditions, the portal TAA collected by dissection at 30 and 60 min after oral yogurt consumption was significantly higher than that after original milk administration. The portal TAA concentrations in blood collected at 90, 120, and 240 min after administration of yogurt or original milk were almost the same [69]. Almost the same results were obtained in an experiment in which a portal vein catheter was introduced in rats to measure changes in portal TAA concentrations over time in the same individuals (unpublished data). In another study comparing original milk and yogurt produced by a different fermentation starter, the portal TAA concentration was significantly higher 30 min after yogurt than the original skim milk administration and was comparable after 60 and 90 min [70]. It has been shown that dairy beverages stabilized in an acidified state have better protein absorption compared with non-acidified dairy beverages [71]. In our study [69], similar to fermented milk, unfermented milk acidified by adding lactic acid had higher TAA absorption compared with the original raw milk. These results suggest that the presence or absence of acid coagulation of milk proteins when mixed with gastric juice has a large impact on protein digestion/absorption. This is supported by the finding in [69] that when gastric juice was depleted using a proton pump inhibitor, amino acid absorption improved 15–30 min after raw milk ingestion. On the other hand, proton pump inhibitors do not affect amino acid absorption after ingestion of acidified milk but reduce it after ingestion of fermented milk [69]. Casein protein may be more highly digestible by pepsin in fermented milk than in raw milk in the stomach [70]. These results indicate that casein protein in fermented milk is partially digested by lactic acid bacterial fermentation, potentially contributing to its reduced resistance to pepsin.

Thus, the apparent digestion and absorption rates of protein can be faster or slower in yogurt than in its unfermented origins, such as raw milk, depending on the manufacturing conditions. However, if fluidity is ensured by avoiding fermentation conditions that result in firmness or by adding a low-viscosity process after fermentation, fermentation should substantially improve the digestion and absorption of proteins. For example, in practical terms, fermented products with reduced viscosity, such as drinking yogurt, are presumed to be advantageous in terms of protein absorption. In addition, increasing the proportion of whey protein in raw material may also be effective in creating yogurt with a higher protein concentration and reduced viscosity [72]. However, processing such as heating, separation, desalting, drying, and storage conditions, such as temperature, humidity, and duration, can alter the state of milk proteins (crosslinking, saccharification, etc.) [73,74,75] and have unintended effects on its digestion and absorption properties. For example, Trommelen et al. tested the hypothesis that caseinate increases postprandial blood amino acid levels more than micellar casein or crosslinked caseinate because of its superior water solubility, but the obtained results were the opposite of their hypothesis [76]. Therefore, it will be necessary to assess the effects of lactic acid bacterial fermentation on the digestion and absorption of milk proteins in more detail via precise studies that not only control physical properties, such as viscosity but also clarify the processing history of the original dairy ingredients.

## 3. The Benefits of Yogurt Protein Intake on Skeletal Muscle Health

### 3.1. Traditional Evidence

The nutritional value of yogurt as a protein source has been investigated since the 1970s; Rasiac et al. showed in vitro that lactic acid bacterial fermentation of milk may increase its biological value [13]. In addition, studies in growing roosters [77] and rats [78] showed that diets containing yogurt increased body weight per unit of protein intake more than diets containing unfermented milk, and thus, the protein in yogurt has higher availability than that in unfermented milk in vivo. Weight gain during growth is accompanied by enlargement of the musculoskeletal system. Therefore, yogurt is presumed to be more advantageous in increasing skeletal muscle compared with the original raw milk. However, until recently, the acute or longitudinal benefits of ingesting protein from yogurt on skeletal muscle have not been investigated in detail.

### 3.2. Acute Effects of Yogurt Ingestion on Skeletal Muscle

Recently, we used an in vivo deuterium biolabeling method for alanine via intraperitoneal heavy water administration to analyze plantaris MPS up to 4 h after ingestion of yogurt, unfermented milk, or acidified unfermented milk. The results showed that the incorporation of deuterium-labeled alanine into muscle protein (i.e., MPS for 4 h) was greater after ingestion of yogurt than unfermented milk or acidified unfermented milk [69]. We also examined postprandial MPS after 30, 60, 90, 120, and 240 min ingestion of these test dairy products using a bolus dose of stable isotope-labeled phenylalanine. As a result, we confirmed that MPS is lower not only after unfermented milk but also after acidified unfermented milk ingestion than after yogurt consumption over the entire measured time [69]. In addition, in a study using yogurt fermented by another lactic acid bacterium, postprandial MPS was increased by yogurt than the original unfermented milk, too [70]. Looking at these reports in more detail, the concentration of blood amino acids, such as leucine, was higher in fermented milk and acidified unfermented milk than in unfermented milk at 30 and 60 min after ingestion [69,70]. This is consistent with the results that MPS is higher at 30 min after ingestion of fermented milk and acidified unfermented milk than of unfermented milk. On the other hand, after ingesting yogurt, MPS tended to increase even at 120 min after ingestion, and this increase in MPS was more sustained than after ingestion of the other test products [69]. This sustained increase in MPS after yogurt consumption did not correspond to changes in blood amino acid or insulin concentrations [69], and thus, another mechanism may be involved in the increased MPS after yogurt ingestion.

We also reported that yogurt consumption increases phosphorylation of p70S6K and 4E-BP1, targets and activation markers of mTORC1, compared with unfermented milk consumption [70]. The difference in mTORC1 activation levels after yogurt versus unfermented milk consumption is likely due, at least in part, to increased blood leucine rather than insulin secretion since Akt phosphorylation levels were comparable and Sestrin2 phosphorylation levels were different [70]. It is interesting to note that the blood amino acid levels and mTORC1 signaling activation tended to be increased when the supernatant was partially removed by centrifugation after lactobacillus fermentation. This supernatant would contain lactic acid and other water-soluble metabolites produced by lactobacillus, such as organic acids, free amino acids, and short peptides [13]. Although these water-soluble low-molecular compounds may be unique functional components of yogurt, their impact on protein absorption and accompanying MPS would not be dominant. Intake of milk protein from quark, which is nonfat milk fermented by *Lactococcus lactis* (i.e., not yogurt exactly), has been shown to increase MPS in both non-exercising and exercising legs of healthy elderly men (average age 73 years) as much as or more than that in younger men (average age 24 years) [79]. In general, the elderly have a weakened protein synthesis response to protein intake and exercise, which is called anabolic resistance [52,80]. Therefore, this result might suggest that lactic acid bacterial fermentation enhances the MPS-stimulating effect of milk proteins in quark, especially in the elderly. However, in this study, the subjects were administered 30 g of protein, which is more than the amount at which anabolic resistance is observed (approximately under 20 g) [81]; thus, it will be necessary to conduct a study using a lower dosage. Finally, the beneficial effect of acute ingestion of fermented milk, including yogurt, on MPS in humans needs further verification in clinical studies using an appropriate placebo.

### 3.3. Longitudinal Effects of Yogurt Ingestion on Skeletal Muscle

Despite the limited evidence, the above results suggest an advantage of yogurt compared with raw milk with respect to protein absorption and short-term MPS enhancement. Thus, long-term consumption of yogurt is expected to have a favorable effect on skeletal muscle mass. However, although several studies have evaluated the complex health benefits of yogurt, no studies have adequately proven the hypothesis that longitudinal yogurt consumption is beneficial on skeletal muscle maintenance and/or increase.

For example, two studies focused on the weight loss effects of yogurt. First, Zemel et al. conducted a 12-week weight loss program in obese, healthy older men (18–50 years old) involving an energy-restricted diet of 500 kcal/day [82]. The subjects were assigned to two groups: one receiving approximately 170 g of nonfat yogurt and the other a gelatin dessert (10 kcal) three times a day. The overall protein/fat/carbohydrate balance of the diets was normalized in each group (however, calcium intake was 500 mg/day higher in the yogurt group) [82]. The weight loss program reduced body weight, including fat-free mass (FFM, primarily reflecting muscle mass) and fat mass, in both groups. Body weight and body fat were reduced more in the yogurt group than in the placebo group, but no such effect was seen on FFM, suggesting that yogurt intake may be effective in maintaining skeletal muscle during weight loss. However, the authors note that the increased priority of fat burning due to increased calcium intake is one factor potentially explaining these results. Thus, the usefulness of yogurt as a protein source remains unclear. Thomas et al. conducted a 16-week weight loss program combining energy restriction (protein intake was maintained) and resistance training (RT) in overweight women [83]. At this time, subjects were assigned to two groups: one was offered yogurt with 10 g of protein after RT, and the other was offered carbohydrates with the same caloric content. Similar to Zemel et al.’s study [82], the weight loss program resulted in weight loss in both groups due to a reduction in fat; on the other hand, FFM was increased in both groups, but there was no additional gain in FFM by yogurt ingestion. This increase in FFM should have been due to the addition of RT to the weight loss program. Therefore, the effect of yogurt may have been masked by the greater effect of RT on FFM maintenance during caloric restriction.

Yogurt has also been used in studies targeting weight gain in underweight individuals and skeletal muscle maintenance in older adults. Bridge et al. conducted two whole-body RT sessions per week and plyometric training once per week in young men who did not undergo training for 12 weeks prior [84]. During the training periods, subjects were administered Greek yogurt or pudding containing isocaloric carbohydrates three times per day on the training day (20 g protein per yogurt serving) or twice per day on the days of no training (15 g protein per yogurt serving) [84]. This training program resulted in an increase in FFM and an increase in maximal muscle strength in both intervention groups. Furthermore, yogurt consumption during the program resulted in higher FFM, increased maximal muscle strength, and decreased body fat percentage compared with placebo pudding consumption, while weight gain was comparable between the two groups. This increased FFM with improved body composition is similar to the effect of yogurt intake during weight loss. Bagheri et al. reported results from healthy older men (average age 68 years) who underwent a whole-body RT program three times a week for 8 weeks [85]. The subjects were administrated Icelandic yogurt containing 18 g of milk protein (traditional concentrated low-fat fermented milk, similar to Greek yogurt, but fermented by *Lactococcus* spp. and *Lactobacillus casei*) or pudding containing isocaloric carbohydrates once daily, immediately after training or at the same time on non-training days. The RT program resulted in increased FFM and muscle strength in both groups. Furthermore, Icelandic yogurt consumption provided additional benefits, including higher FFM, increased maximal muscle strength, and greater weight loss, compared with placebo pudding consumption. Furthermore, these benefits of yogurt consumption were partially caused by increased levels of positive regulators of skeletal muscle mass, such as IGF-1 and follistatin, and by decreased levels of negative regulators, such as TGF-β, GDF15, activin A, and myostatin. However, as mentioned in the rebuttal report [86] to the study by Bagheri et al. [85], an appropriate placebo was not established in terms of validation of yogurt’s benefits. In other words, although a placebo control in terms of calories was established in the above previous studies, studies employing the same amount of protein as yogurt, or more specifically the same amount of protein from unfermented original milk, as a placebo are necessary.

## 4. Relationship between Milk Protein and the Biological Regulatory Functions of Yogurt Based on Lactobacillus Fermentation

### 4.1. Gut–Muscle Axis

We have addressed the usefulness of consuming milk protein from yogurt by discussing the absorption of milk protein in yogurt and its effect on skeletal muscle. On the other hand, recently, the relationship between the biological regulatory functions of yogurt described in Section 1.2 and skeletal muscle has attracted attention. There has also been a focus on the relationship between skeletal muscle and the intestinal microbiota, the so-called gut–muscle axis, based on the probiotic effects of viable bacteria in fermented food and/or the prebiotic effects due to dead food bacterial body, metabolites, polysaccharides, and other secreted products.

Studies investigating the relationship between intestinal microbiota and muscle mass have reported that several lactic acid bacteria are correlated with muscle mass [87]. In a recent review, Marco Castro et al. indicate that age-related abnormalities in the intestinal microbiota and/or increased intestinal permeability may lead to sarcopenia via chronic inflammation and decreased protein digestion [88]. In general, it has been suggested that chronic inflammatory conditions due to aging, obesity, and chronic disease can induce resistance to anabolic stimuli, including insulin resistance, leading to sarcopenia [89]. After yogurt consumption, anti-inflammatory effects may be exerted via lactobacillus metabolites and products.

### 4.2. Probiotic Effects

Recently, there has been an increasing number of studies evaluating the effects of probiotics, such as lactic acid bacteria and bifidobacteria, in aged or aging-induced mice, and there is growing evidence that sarcopenia can be prevented by administering probiotics [90,91,92,93]. In addition, a review and meta-analysis of human randomized controlled trials reported by Prokopidis et al. showed that probiotic supplementation enhanced both muscle mass and strength [94]. However, it is important to clarify the characteristics of the probiotics suitable for maintaining and strengthening skeletal muscle. The benefits of probiotics on skeletal muscle are related to an increase and/or maintenance of bacterial populations that produce short-chain fatty acids, such as butyrate, in the intestinal flora [87,88]. The probiotic effects of *Streptococcus thermophilus* and *Lactobacillus delbrueckii* subsp. bulgaricus, which are used for yogurt production, on skeletal muscle mass and function have not been established; however, *L. delbrueckii* supplementation contributed to increased production of short-chain fatty acids in the intestines of piglets [95]. Thus, there may be certain species suitable for producing yogurt that also improve skeletal muscle conditions.

### 4.3. Effects of Prebiotics and Other Ingredients

In addition to probiotic effects, the contribution of ingredients involved in lactobacillus fermentation is also interesting. For example, milk proteins from lactic acid bacterial fermentation have been shown to not only increase digestibility but also increase the production of bioactive peptides that differ from those in the original milk [57]. In addition, observational studies have shown that yogurt consumption is linked to a decreased risk of type 2 diabetes [96,97]. Recently, it was demonstrated that this effect of yogurt is due, at least in part, to improved glucose metabolism via supplementation with branched-chain hydroxy acids (BCHAs), the concentration of which increases in yogurt as a result of lactobacillus metabolism [98]. Our group reported that α-hydroxy isocaproic acid, a BCHA, prevents atrophy of C2C12 mouse myotubes due to inflammatory cytokine exposure [99], and BCHAs may be a key ingredient to examine regarding the health benefit of yogurt on skeletal muscle. In a study focusing on the anti-inflammatory effects of polysaccharides produced by lactobacillus fermentation of Caspian yogurt containing rich polysaccharides compared with a normal diet in 18-month-old mice for 8 weeks, yogurt treatment reduced TNFα and increased IGF-1 levels in skeletal muscle, and thereby improved muscle mass accompanying increased phosphorylation of mTORC1 and p70S6K [100].

Modification of the gut–muscle axis via lactic acid bacteria and/or the metabolites produced during their fermentation could have a positive effect on muscle mass and strength, especially in elderly and chronic disease patients. This new viewpoint is worth considering for additional benefits of obtaining protein from yogurt.

## 5. Current Limitations and Future Prospects

Currently, many reports suggest that the rate of gastric transit is particularly important for the rate of protein absorption, and thus physical properties, such as the particle size and agglomeration state of food proteins prior to ingestion, contribute to protein digestion/absorption [20,66,67,68,69,70,101]. Therefore, although consuming milk protein via yogurt is considered to be advantageous over raw milk in terms of protein absorption, the manufacturing process and the physical properties of the product need to be considered to maximally reap the benefits [102]. In addition, consuming yogurt as a source of protein can be effective for maintaining and/or increasing skeletal muscle due to the protein availability and bioregulatory functions of yogurt.

No randomized controlled trial using an appropriate placebo group in terms of yogurt validation has been conducted. However, such studies need to be conducted on a large number of subjects, which may be too difficult to conduct randomized controlled trials to prove differences, including our group. Indeed, animal proteins, including milk protein and plant proteins, such as soy protein, are expected to have different effects on skeletal muscle during long-term consumption due to the magnitude of differences in their digestion and absorption characteristics, and many studies have been conducted. However, the differences among these proteins are not completely consistent among trials [103]. Therefore, it may be more difficult to investigate the differences between yogurt and original raw milk as a placebo. However, if more clinical trials using yogurt are conducted in the future, we can expect that the usefulness of yogurt in maintaining and increasing skeletal muscle mass will be demonstrated by meta-analyses and other methods. Of course, parallel efforts are needed to deepen our understanding of the complex functions of yogurt via preclinical research.

In addition, this review has focused on yogurt in the strict definition of the term, although it also included a few studies on non-yogurt fermented milk. Further efforts in this arena include the development of novel fermented foods that contribute to skeletal muscle health, resulting in an extended healthy life expectancy, by freely combining various protein ingredients and types of food bacteria from the perspective of sustainable ingredient supply.

## 6. Conclusions

**Figure 2:**
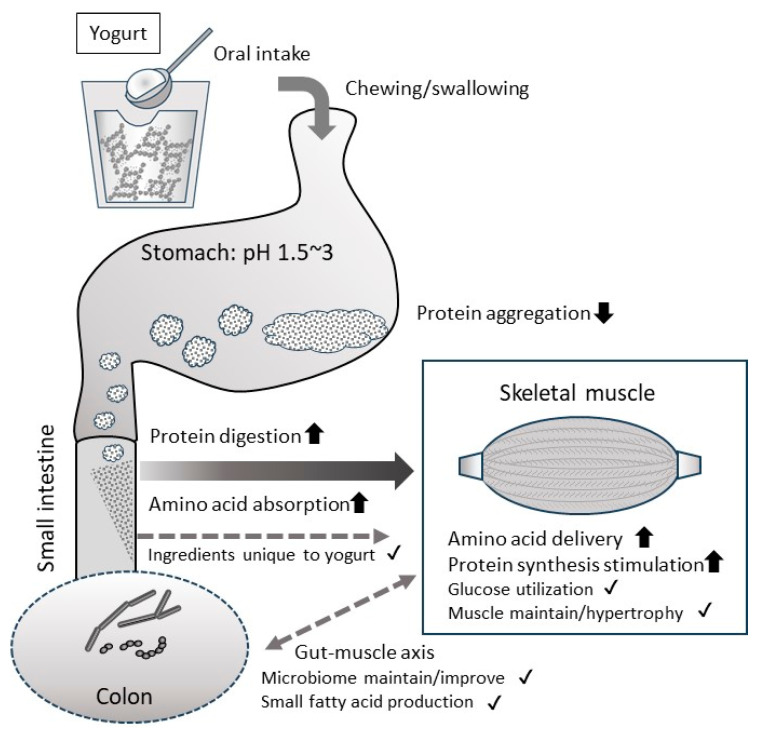
Expected health benefits of consuming milk protein as yogurt on skeletal muscle. The milk proteins in yogurt are soft, acidified gel, so they will not aggregate tightly in the stomach. This will facilitate mixing with gastric juice through gastric peristalsis, pre-digestion with pepsin, and transport to the small intestine. Milk proteins that reach the small intestine are neutralized and then rapidly digested by pancreatin. Therefore, overall, milk protein intake as yogurt can be expected to improve the digestibility/absorption of proteins. However, care must be taken as it largely depends on the physical properties of the yogurt. Because milk protein in yogurt is highly digestible and absorbable, it is expected that the supply of amino acids to skeletal muscles will improve, leading to increased skeletal muscle protein synthesis. The unique ingredients and bacterial body contained in yogurt can have a positive effect on skeletal muscles via the gut–muscle axis and so on. By accumulating these positive effects, we can expect to maintain and increase skeletal muscle mass over the long term. However, most of these benefits have not yet been definitively proven and require further research. The arrows in the figure indicate benefits for which some evidence has been shown. The checkmarks in the figure indicate benefits that can be expected based on previous knowledge, although there is no direct evidence.

A review of the present research suggests that among the dairy foods considered a high-quality protein source, yogurt may be a particularly good source for increasing muscle mass. Figure 2 summarizes the expected benefits of consuming milk protein from yogurt, leading from this review. Since it especially affects absorption properties due to differences in physical properties, it is important to optimize the manufacturing conditions and the form of the product, such as liquid or semi-solid, and so on. Also, the properties of the bacteria used in fermentation may also play a role in yogurt’s benefits. However, since there is little solid evidence for this benefit of yogurt, especially in humans, further efforts are needed.

## Abbreviations

mTORC1: mechanistic target of rapamycin complex 1, PI3K: phosphatidylinositol-3 kinase, Akt/PKB: protein kinase B, Glut4: glucose transporter type 4, FOXO: Forkhead box O, p70S6K: Ribosomal protein S6 kinase beta-1, 4E-BP1: Eukaryotic translation initiation factor 4E-binding protein 1, MuRF1: muscle RING finger protein-1, GLP1: glucagon-like peptide 1, GIP: gastric inhibitory polypeptide, GATOR2: GTPase-activating protein activity toward Rags 2, tRNA: transfer RNA, SDS-PAGE: sodium dodecyl sulfate-polyacrylamide gel electrophoresis, IGF1: insulin-like growth factor 1, TGF-β: transforming growth factor β, GDF15: growth differentiation factor-15.
